# Uncommon Site Presentation: A Case Report of Extraparotid Warthin’s Tumor

**DOI:** 10.7759/cureus.61734

**Published:** 2024-06-05

**Authors:** Nurul Atikah Zakaria, Irise Hoi Khin Chen, Enci Yong, Mazlinda Mahadzir

**Affiliations:** 1 Otorhinolaryngology-Head & Neck Surgery, Hospital Putrajaya, Putrajaya, MYS; 2 Radiology, Hospital Putrajaya, Putrajaya, MYS

**Keywords:** warthin’s tumor submandibular gland, lateral cystic neck mass, benign neoplasm submandibular gland, extraparotid warthin’s tumor, warthin’s tumor

## Abstract

Warthin’s tumor, also known as adenolymphoma or papillary cystadenoma lymphomatosum, is a benign tumor almost exclusively found in the parotid gland and is the second most common type of benign parotid tumor. Its manifestation as an extraparotid lesion is rare, with a low incidence in the submandibular gland. In this context, we present a case of Warthin’s tumor of the submandibular gland in a 66-year-old man who presented with a painless lateral cystic cervical mass. This case highlights the clinical and radiological evidence of an uncommon extraparotid tumor location, with the diagnosis becoming evident only after the enucleation of the mass. Despite the rarity of extraparotid Warthin’s tumor and its potential variation in location, the authors recommend considering Warthin’s tumor of the submandibular gland in the differential when assessing lateral cervical masses.

## Introduction

Warthin’s tumor predominantly develops in males, with its peak incidence in the sixth to seventh decade [1]. It is the second-commonest benign tumor of the parotid gland, frequently associated with smoking, and has a rare tendency toward malignant transformation [1]. The exact pathogenesis of this tumor is not entirely known, but it was proposed that Warthin’s tumor develops from salivary duct inclusions within the intraparotid lymph nodes [1]. It commonly involves the tail of the parotid gland and typically grows slowly with a predilection for bilateral presentation [2]. The occurrence of extraparotid Warthin’s tumor is unusual, with an incidence of approximately 2.7-12% of cases and commonly found in the peri-parotid area as well as the oral cavity, oropharynx, and larynx [3]. A large retrospective study conducted by Xu W et al. revealed that out of 1084 cases of Warthin’s tumor, the commonest location was in the parotid gland, followed by intra- or peri-parotid lymph nodes (n=13), upper neck (n=10), submandibular gland (n=4) and upper lip (n=1) [4].

The common presenting symptom is a painless, slow-growing mass over the cervical region, with a 10-15% incidence of bilaterality [1]. In this type of tumor, ultrasonography may highlight a well-defined heterogeneous, hypoechoic lesion with a septation and cystic component [1]. Meanwhile, computed tomography may demonstrate a well-circumscribed, homogenous hypodense lesion with a clear edge, potentially containing cystic and solid components [4]. Histologically, Warthin’s tumor exhibits epithelial and lymphoid components, showcasing a bilayered epithelial lining composed of inner columnar eosinophilic or oncocytic cells surrounded by smaller basal cells [5]. In the management of Warthin’s tumors, some authors advocate surgical excision. However, due to its slow-growing nature and according to the literature, malignant transformation of Warthin’s tumor is extremely rare and occurs in about 0.3% of cases [1]. Therefore, conservative measures are also considered an option. Herein, we present a case of Warthin’s tumor of the submandibular gland in an elderly man, where a simple cervical cyst was suspected at the initial presentation.

## Case presentation

A 66-year-old gentleman presented with a painless left cervical swelling that has progressively increased in size over the past two years. There was no evidence of obstructive symptoms, such as dysphagia, breathlessness, noisy breathing, and hoarseness. There was no fever, no apparent constitutional symptoms, and no history of recent neck trauma or injury. He is a non-smoker, and he denied having contact with tuberculosis. External examination of the neck revealed a left lateral cervical mass measuring 3 x 3 centimeters (cm). The mass was located cranially at the left angle of the mandible, extending caudally to the level III cervical region. On palpation, the mass was soft in consistency, non-tender, mobile, and non-ballotable. The transillumination test was positive, and there were no other palpable cervical lymph nodes. The oropharynx and other ENT examinations were unremarkable. Endoscopies of the nasopharynx and larynx were normal.

**Figure 1:**
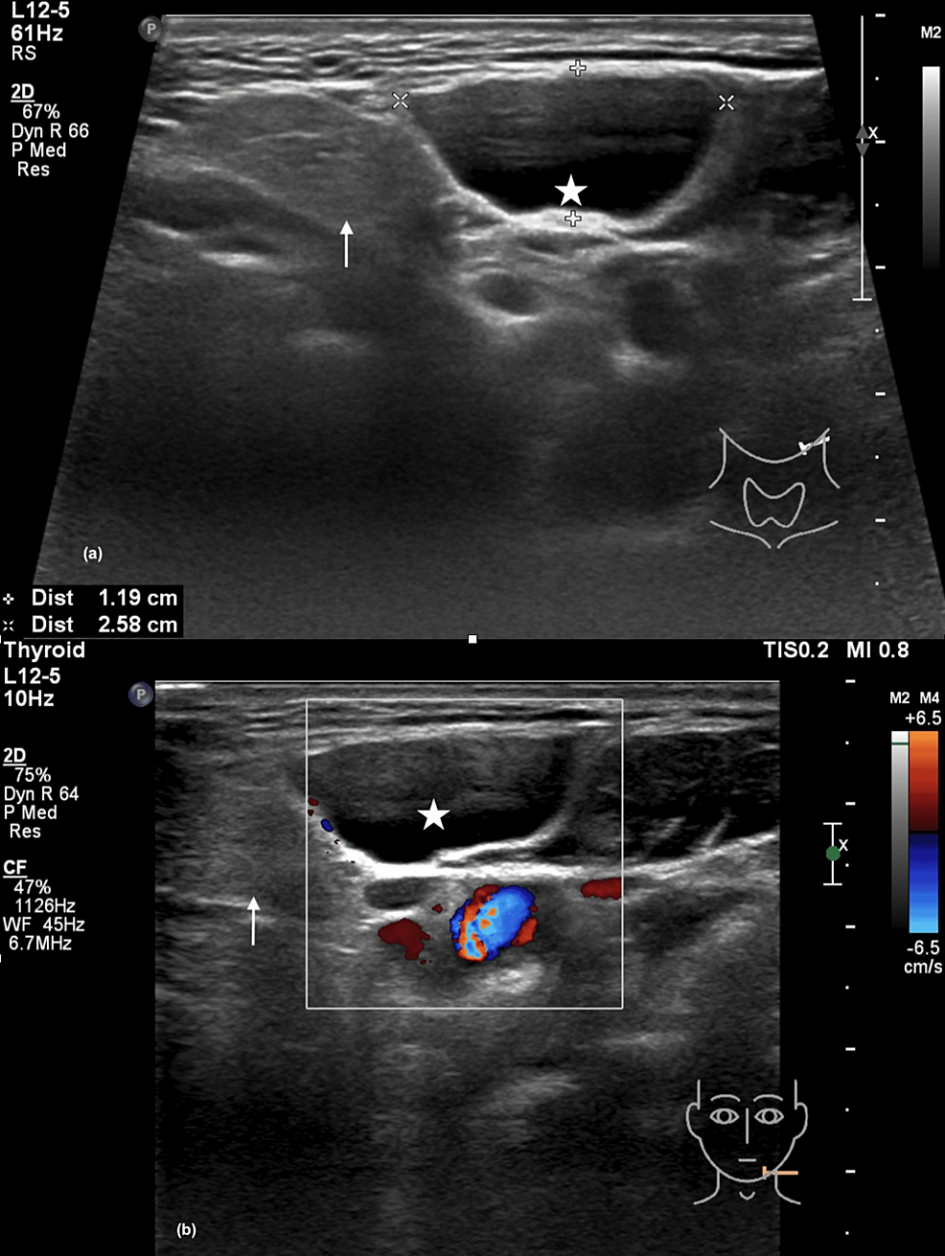
Ultrasonography of the neck (a) An anechoic mass (star) with well-defined margin and thin walls, located anteromedial to the left sternocleidomastoid muscle, and a normal submandibular gland (arrow); (b) the mass demonstrated no internal vascularity on color doppler.

Ultrasonography of the neck revealed a 1.0 x 2.6 x 3.8 cm oval anechoic lesion with well-defined margins and thin walls located anteromedial to the left SCM muscle. The lesion showed septation with no calcification, debris, or vascularity within (Figure 1).

**Figure 2:**
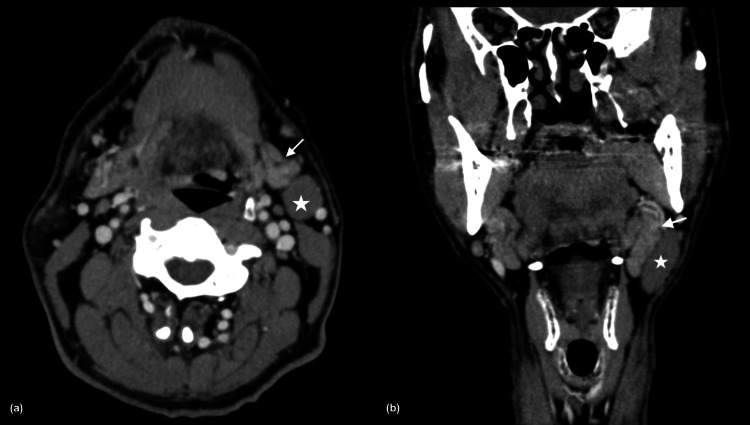
Contrast-enhanced computed tomography of the neck (a) A well-circumscribed, homogenous hypodense lesion (star) at the left lateral cervical region and a normal left submandibular gland (arrow); (b) a distinct separation from the tumor on the coronal.

A contrast-enhanced computed tomography (CECT) demonstrated a well-circumscribed homogenous hypodense lesion at the left lateral cervical region, measuring approximately 2.2 x 2.1 x 3.7 cm, and this mass was located anterior to the left SCM muscle, with no clear fat plane between its anterior and medial border of the muscle. The left submandibular and parotid glands were normal, and there was a clear fat plane seen with the left submandibular gland (Figure 2).

Fine needle aspiration for cytology (FNAC) examination revealed only cyst content, with no evidence of atypia or malignant cells.

**Figure 3:**
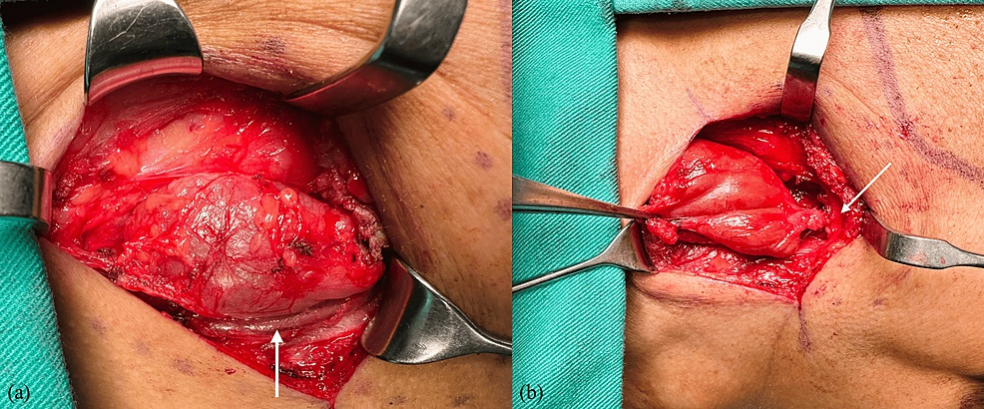
Intraoperative picture (a) A well-dermacated cystic mass is located superficial to the left sternocleidomastoid muscle. The left external jugular vein (arrow) was seen posterior to the mass; (b) Cranially, the mass was abutting the submandibular gland (arrow) with no clear plane.

**Figure 4:**
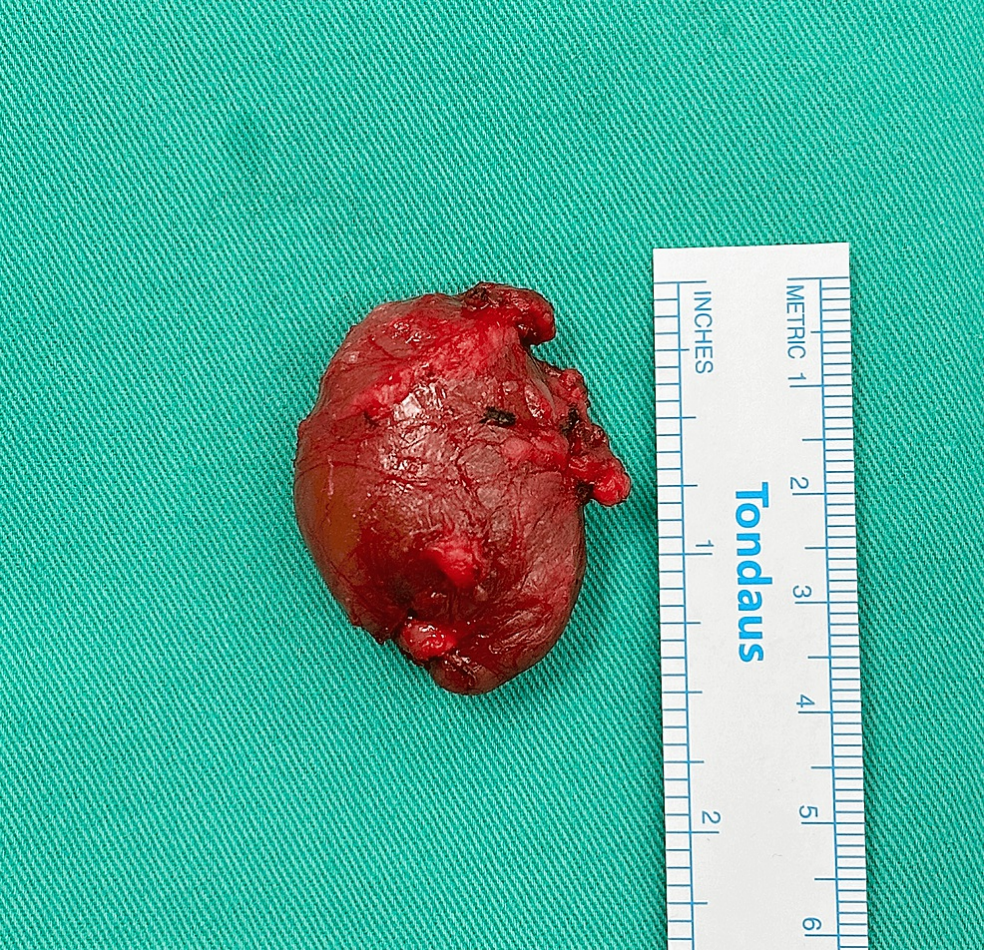
Macroscopic picture of the cervical mass Cystic mass measuring 4 x 4 cm, surrounded by a thin capsule.

Correlating these features of the left lateral cervical mass located at the level of the SCM muscle and its cystic nature, with no association with surrounding glands, suggests a distinct clinical presentation of a possible left cervical cyst. The patient underwent mass enucleation via a transcervical approach. Intraoperatively, the mass measured approximately 4 x 4 cm, located superficial to the left SCM muscle. Cranially, the mass was abutting the submandibular gland with no clear plane, and caudally, it extended to the middle third of the SCM muscle. The left external jugular vein was seen located posterior to the mass (Figures 3, 4).

Given the left lateral cervical mass and its proximity to the left submandibular gland with no clear plane, an intraoperative diagnosis of a benign neoplasm of the submandibular gland was made. The decision was made to excise the cystic mass along with a cuff of submandibular gland tissue, where the mass was ligated and removed in toto.

**Figure 5:**
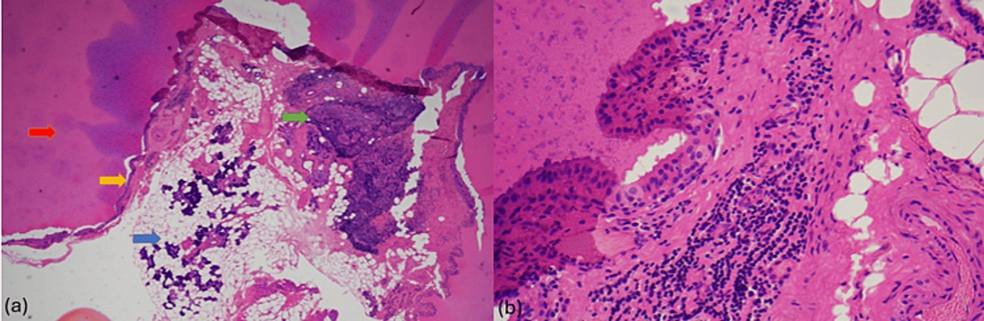
Histopathological examination (a) The cyst is lined by double layers of oncocytic cells (yellow arrow) and filled by eosinophilic secretion (red arrow). A few lymphoid aggregates were seen adjacent to the largest cyst (green arrow), and benign salivary gland tissue was included (blue arrow) (4x magnification); (b) The cells exhibited central bland nuclei with small nucleoli in abundant granular eosinophilic cytoplasm (40x magnification).

The histopathological examination demonstrated a large cystic lesion lined by a double layer of oncocytic epithelium and round central nuclei with small nucleoli in an abundant granular eosinophilic cytoplasm. Benign salivary tissue was included, with no nuclear atypia or malignancy (Figure 5). A Warthin’s tumor of the submandibular gland was confirmed. Upon follow-up with the patient three months post-surgery, he was well. 

## Discussion

Warthin’s tumor commonly and almost exclusively occurs in the parotid gland. Hence, its incidence as an extraparotid tumor is rare. The occurrence of submandibular gland Warthin’s tumor is only about 0.6% to 1.3% [2]. There is a strong association between smoking and Warthin's tumor, and evidence suggests that the risk is eight times higher in smokers compared to non-smokers [2]. Although smoking is not associated with this reported case, some authors have hypothesized that the pathogenesis of Warthin’s tumor may result from its origin in heterotrophic salivary parenchyma or ductal inclusions in the intra- and peri-glandular lymph nodes during the embryogenic development into parotid glands [6]. In this case, the authors postulate that the same hypothesis may have occurred in the submandibular gland despite its scarcity of cases, and other factors such as genetics and the environment may also impact these inclusions, giving rise to Warthin’s tumor of the submandibular gland.

Despite the limitations of ultrasonography in the imaging of the neck, it is easily accessible, cost-effective, and non-invasive and enables the authors to confirm the mass was cystic, allowing the early exclusion of vascular tumors. Often, in cystic cervical masses, the mass is soft and compressible and may exhibit an occasional transmitted pulsation during clinical examination. Therefore, an initial ultrasound is beneficial before FNAC. Nevertheless, CECT offers more benefits in this case, as it allows precise identification of the exact location, origin, and extension, which is imperative for surgical planning. Specifically, in this case, a simple cervical cyst, inflammatory cyst, or branchial cleft cyst were among the differential diagnoses; hence, CECT may also aid in confirming the possibility of a branchial fistula or collection and in excluding other possible diagnoses, such as cystic neoplasm of the salivary glands.

The mass was located in the left lateral cervical region, lying superficial to the SCM muscle, accompanied by a non-ballotable swelling and an intraoral examination showing no raised floor of the mouth. Conversely, the CECT demonstrated a well-circumscribed homogenous hypodense lesion at the left lateral cervical region, with no clear fat plane between the anterior and medial borders of the SCM. In addition, there was no radiological evidence supporting a submandibular gland neoplasm, as the gland appeared normal with a distinct separation from the tumor. Based on its location in the CECT and an anechoic mass in ultrasonography as complementary evidence, a cervical cyst was deemed a more probable diagnosis. Considering the CECT findings, the authors overlooked Warthin’s tumor of the submandibular gland as a potential diagnosis.

In the management of cervical masses, FNAC is the first tool of investigation to confirm the diagnosis, as it is minimally invasive and allows the exclusion of malignancy. However, the role of FNAC in cystic masses is limited due to their hypocellular content. Fernandes H. et al. found that only 4 out of 10 cases of cystic cervical masses were suitable for histopathologic evaluations. They suggested that this limitation could be due to a sampling error during the procedure, where only the cystic area was aspirated. Consequently, they recommended the excision of cystic masses for a definite diagnosis [2]. Similarly, in this reported case, the authors chose excision of the cervical mass as a diagnostic and therapeutic approach when the FNAC results were inconclusive. Interestingly, during the surgery, the authors discovered that the cervical mass was abutting the left submandibular gland with no clear plane, contrary to the pre-operative CECT evaluation. At this point, the diagnosis of a submandibular cyst was highly suspected. However, the decision was made to only excise the cystic mass with a cuff of the submandibular gland tissue, as submandibulectomy was not feasible due to the absence of written consent.

Warthin’s tumors are traditionally managed by surgical excision, and Warthin’s tumor of the parotid gland requires a superficial parotidectomy. In recent years, Quer M et al. conducted a comprehensive literature review, which included 141 articles on the management of Warthin’s tumors. They advocate that due to its slow growth rate, mild clinical symptoms, and advanced age of patients, active surveillance has become widely implemented. They also propose four indications for surgery, including an uncertain diagnosis, cosmetic concern, intolerable symptoms such as pain, and the patient’s wish. The extent of surgery was also discussed, and it is evident that the current trend is to minimize the extent of resection to extracapsular dissection or enucleation of the tumor, as the risk of malignant transformation is extremely rare [7]. For this reason, the authors believe that the enucleation of Warthin’s tumor, in this case, is sufficient.

## Conclusions

Despite the rarity of extraparotid Warthin’s tumor and its potential variation in location, even serial investigations may be inconclusive due to the tumor’s nature. This may result in inconclusive FNAC results and inconsistent radiological findings, leading to suboptimal pre-operative planning. Therefore, the authors recommend keeping Warthin’s tumor and other benign neoplasms of the submandibular gland on the differential when assessing lateral cervical masses. Fortunately, in this case, the final diagnosis was Warthin’s tumor of the submandibular gland, making complete enucleation of the tumor adequate.
